# Nanoparticles carrying paclitaxel and anti-miR-221 for breast cancer therapy triggered by ultrasound

**DOI:** 10.1038/s41420-023-01594-9

**Published:** 2023-08-15

**Authors:** Libo Zhang, Zhen Ren, Jinhui Lü, Xinhai Mo, Jie Lin, Ya Li, Wenjing Ma, Pengfei Liu, Yajing Shen, Qian Zhao, Lu Qian, Xiaoxin Cheng, Zuoren Yu, Bo Zhang

**Affiliations:** 1 Department of Ultrasound Medicine, Shanghai East Hospital, Nanjing Medical University, 150 Jimo Road, Shanghai, China; 2 Research Center for Translational Medicine, Shanghai East Hospital, Tongji University School of Medicine, Shanghai, China; 3 School of Basic Medical Sciences, Dalian Medical University, Dalian, China

**Keywords:** Breast cancer, Drug delivery

## Abstract

Nanomaterials have been well demonstrated to have the potential to be used for tumor cell-targeted drug delivery. Targeted inhibition of miR-221 was proved to promote the sensitivity of triple genitive breast cancer (TNBC) cells to chemo-drugs. In order to improve the chemotherapeutic effect in TNBC, herein, we developed a novel kind of nanoparticles shelled with PLGA and loaded with perfluoropentane (PFP), paclitaxel (PTX), and anti-miR-221 inhibitor, which was named PANP. Ultrasound-triggered vaporization of PFP in PANPs was utilized for real-time imaging track of the nanoparticles in vivo. In addition, macrophages were applied for the internalization of PANPs to form RAW-PANP with strong chemotaxis to accumulate around cancer cells. Nanoparticles with different contents did not cause M2 polarization compared with the control group but caused polarization toward M1. We compared the inherent tumor-homing behavior of macrophages containing different contents with that of normal macrophages and no significant abnormalities were observed. After injection into the tumor-burden mice, RAW-PANPs showed enrichment within tumor tissues. Upon the ultrasound cavitation-triggered burst, PTX was released in the tumor. Meanwhile, the release of anti-miR-221 improved the sensitivity of tumor cells to PTX. As a result, RAW-PANPs showed high efficiency in suppressing TNBC cell proliferation in vitro and inhibiting tumor growth and progression in vivo. The treatments did not induce liver, heart, or kidney injury. In conclusion, the current study not only developed a macrophage-carried, ultrasound-triggered, cancer cell-targeted chemotherapeutic system, but also demonstrated a miRNA-based technique to promote drug sensitivity of cancer cells, which holds strong potential to treat patients with TNBC, especially for those suffering drug-resistance. The innovation of this study is to use macrophages to deliver nanoparticles to the tumors and then use ultrasound locally to burst the nanoparticles to release the miRNA and PTX.

## Introduction

Triple-negative breast cancer (TNBC) is characterized by high clonal heterogeneity, frequent metastasis, and poor prognosis [1–3]. Surgery, radiotherapy, and chemotherapy are the only ways to treat patients with TNBC. Unfortunately, drug resistance, side effects, and uncertain therapeutic effects limit the application of radiotherapy and chemotherapy.

Cancer cell-targeted drug delivery and cancer cell-targeted drug sensitizers are considered the most promising approaches to improving the therapeutic effect of anticancer drugs [4–7]. In addition, emerging evidence has indicated the potential of nanomaterials in the application of drug delivery to tumor tissues [8]. Nanoparticles can deliver anticancer drugs into cells in vitro and in vivo in a cell-targeted delivery manner by assembling a specific ligand of target cells to the shell surface of nanoparticles.

Paclitaxel (PTX), as one of the most commonly used first-line chemotherapy drugs in the treatment of cancer patients, including breast, lung, and ovarian cancers, has a significant therapeutic effect on inhibiting cancer cell proliferation [9, 10]. PTX resistance can be observed in TNBC and involves a variety of microtubule-associated proteins [11]. The microtubule-binding protein Tau and PTX form a competitive relationship for microtubules, which attenuates the effect of PTX in promoting microtubule aggregation; in addition, the apoptosis susceptibility (CAS) protein inhibits PTX-induced apoptosis, probably through its reduced blockade of the G2/M phase of the cell cycle and the assembly of microtubule stars [11]. CSE1L/CAS, a microtubule-associated protein, inhibits PTX-induced apoptosis but enhances cancer cell apoptosis induced by various chemotherapeutic drugs [12].

In order to maximize the therapeutic effect of PTX, a kind of nanoparticles carrying PTX (called liposome paclitaxel or EndoTAG-1) [13] has been prepared and successfully applied to patients with TNBC in a phase II clinical trial [14], showing much better therapeutic outcomes than naked PTX. Still, treatment resistance was observed, and the formulation might require optimization. Such a way could be combining PTX with micro RNAs (miRNAs) and macrophages [15]. miRNAs, as non-coding small RNA, are involved in human cancer [16]. It has been reported that the drug sensitivity of cancer cells is closely related to miRNAs. For example, our previous studies identified miR-221 as a key regulator of cellular invasion and chemosensitivity in TNBC [17]. Targeted inhibition of miR-221 by its antisense oligonucleotide (ASO) significantly increased the sensitivity of the TNBC cell line MDA-MB-231 to doxorubicin and cisplatin [18]. A study also reported the involvement of miR-221 in the regulation of PTX sensitivity in TNBC cells [19] and in lung cancer [20]. Therefore, combining an anti-miR-221 inhibitor and anticancer drugs could hold promise to improve the therapeutic effect of chemotherapy and modulate chemoresistance.

Macrophages, as one of the most abundant immune cells in the tumor microenvironment (TME), are closely correlated to tumor development and progression [21, 22]. At the initial stage of tumor development, macrophages inhibit tumorigenesis by activating other immune cells to kill the tumor cells. On the other hand, at the stage of tumor progression, macrophages exhibit an immunosuppressive phenotype by promoting tumor progression and inducing chemoresistance. Macrophages have strong chemotaxis to accumulate around cancer cells, which can be used to develop local delivery approaches to send drugs directly into the tumors. For example, macrophages (RAW 264.7 cells) have been used to internalize doxorubicin-loaded nanoparticles to form a kind of cell bomb to treat cancer cells in vitro or tumor-carrying mice in vivo, showing enrichment around cancer cells and strong therapeutic effect as well [23]. In addition, FA-CD@PP-CpG nanocomposites were shown to display an efficient synergistic therapeutic effect in docetaxel-enhanced immunotherapy for the clinical application of breast cancer [7]. By taking advantage of the liquid-gas phase transition characteristics of perfluoropentane (PFP), PFP can be encapsulated into nanoparticles with doxorubicin, making it possible to track the nanoparticles in vivo by real-time imaging upon ultrasonic stimulation [23]. After accumulation within tumors, phase transition in the nanoparticles can be triggered by ultrasound, resulting in the generation of numerous large microbubbles, damage of the nanoparticles, and local release of doxorubicin [23].

**Fig. 1:**
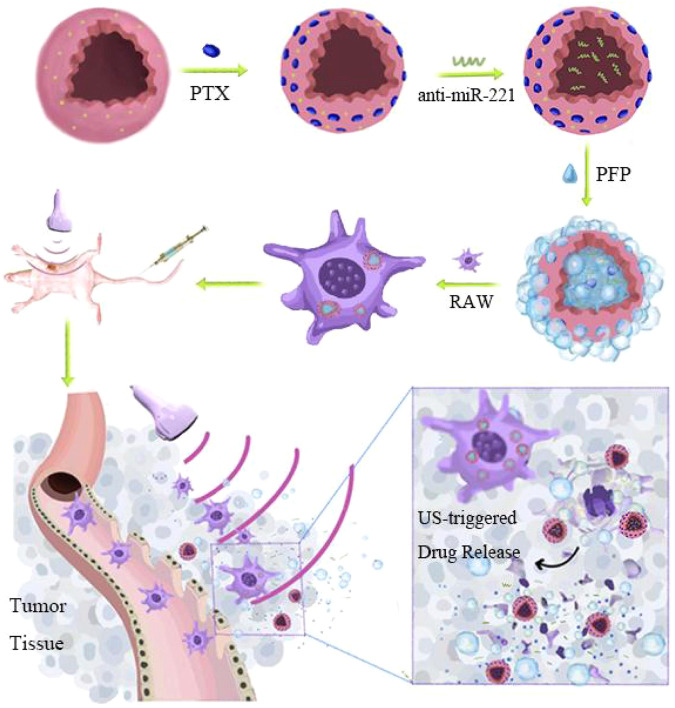
Schematic design of a macrophage-mediated drug delivery system. Perfluoropentane (PFP), paclitaxel (PTX), and anti-miR-221 were loaded into shells to form the nanoparticles with full components (PANPs) and then incubated with macrophages at 37 °C in the cell culture medium to obtain RAW-PANP. After injection into mice through the tail vein, a small fraction of PFP produced small bubbles in response to low-intensity ultrasound, enabling real-time tracking in vivo. Macrophages carrying PANPs accumulated within the tumor tissues. Upon high-intensity ultrasound, the remaining PFP burst, releasing PTX and anti-miRNA.

This study aimed to develop a highly effective approach for the chemotherapy-based treatment of TNBC by preparing a novel kind of nanoparticles shelled with PLGA/lipid and loaded with PTX, PFP, and anti-miR-221 together (which was named PANP) and delivered by macrophages. This strategy could be used to overcome PTX resistance and improve the prognosis of TNBC (Fig. 1).

## Results

### Characterization of PANPs

**Fig. 2:**
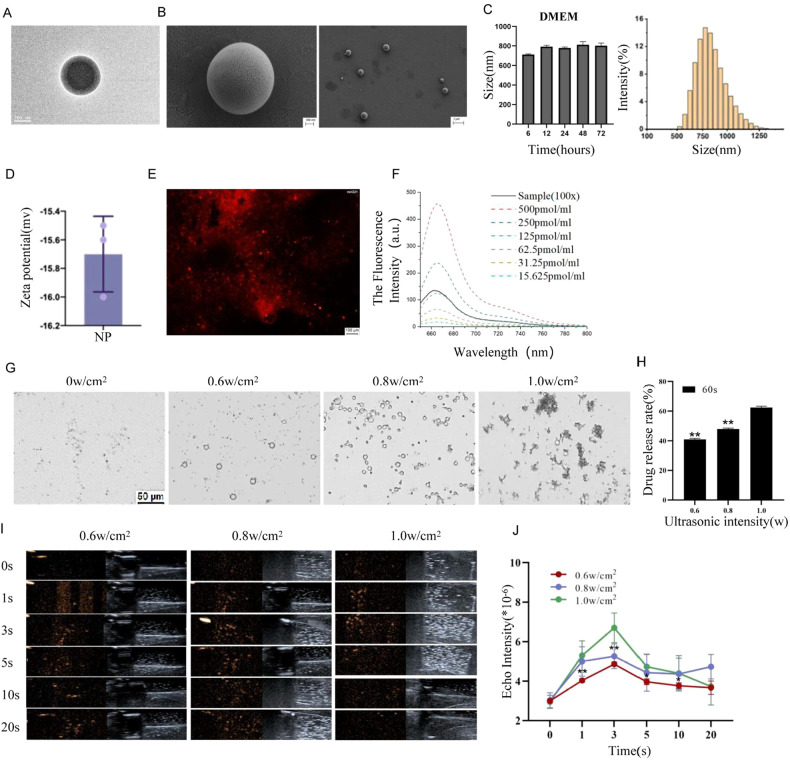
Characterization of PANPs. **A**, **B** Representative images of PANPs by TEM (transmission electron micrograph) (**A**) and PEM (projection electron microscopy) (**B**). **C** Size of PANPs in DMEM at 37 °C. **D** Zeta-potential of PANPs at 37 °C measured using a Zeta dynamic light scattering analyzer. **E** The anti-miR-221 was stained using Cy5 after fluorescence microscopy of the observed images, and its content was detected by fluorescence spectrophotometry (green for the shell and red for anti-miR-221). **F** The Cy5 fluorescence intensity in **E** shows a dose-dependent manner. **G** Representative images of PFP vaporization in PANPs in DMEM by ultrasonic stimulation with intensities of 0, 0.6, 0.8, and 1.0 W/cm^2^. **H** High-performance liquid chromatography (HPLC) analysis of the PTX release from PANPs in DMEM upon ultrasound sonication showing an intensity-dependent manner. **I** Ultrasound images of PANPs in Matrigel before and after sonication with different intensities of 0.6, 0.8, and 1.0 W/cm^2^ for different times from 0 to 20 s. The left figure is an ultrasonography image, and the right is a conventional two-dimensional image. **J** Quantitative analysis of echo signal intensities in **I**. Data are presented as the mean ± SEM (*n* = 3). **P < 0.01.

After preparation of PANP using the double emulsification method, TEM and SEM indicated a regular spherical morphology (Fig. 2A (scale bar: 200 nm) and 2B (scale bar: 100 nm)) with an average diameter from 612.1 ± 25.6 nm at origin to 780.0 ± 40.7 nm after 12 h of culture in DMEM containing FBS at 37 °C (Fig. 2C). The size remained stable in the medium until 72 h. The average surface Zeta potential was −14.23 ± 8.69 mV (Fig. 2D). As shown in Fig. 2E, F, the RFP signals of small RNAs were detected (Fig. 2E), and the fluorescence intensity showed a dose-dependent manner (Fig. 2F). In order to test the vaporization efficiency of the nanoparticles, different ultrasonic intensities (0, 0.6, 0.8, and 1.0 W/cm^2^) were applied. Vaporization of PFP, an increase of the nanoparticle volume, and the formation of gas bubbles were observed after ultrasonic treatment, which indicated that the most effective intensity was 0.8 W/cm^2^ for vaporization and 1.0 W/cm^2^ for burst (Fig. 2G). Representative images of the vaporization of nanoparticles in the culturing medium before and after sonication are shown in Supplementary Fig. S1. In addition, PTX release efficiency upon ultrasound was determined by HPLC analysis, showing an intensity-dependent manner (Fig. 2H). Different concentrations (0, 5, 50, 500, 5000, and 50,000 µg/mL) of PANP were co-cultured with RAW264.7 cells using the CCK-8 method to observe the effect of different concentrations of PANP on the viability of RAW cells (Supplementary Fig. S2).

PANPs were analyzed after embedding in Matrigel to mimic their status in vivo. Contrast-enhanced (a feature available on the Aplio 500 system) ultrasound images before and after sonication with 0.6, 0.8, and 1.0 W/cm^2^ for 0 to 20 s [24] are shown in Fig. 2I. As shown in Fig. 2J, sonication of PANPs for 3 s with 1.0 W/cm^2^ was the best condition for ultrasonic imaging.

NP with different contents did not cause M2 polarization compared with the control group but caused polarization toward M1. M1 macrophages are an antitumor type of macrophages and will not affect the subsequent experiments (Supplementary Fig. S3).

### The tumor-suppressing capability of PANPs in vitro

**Fig. 3:**
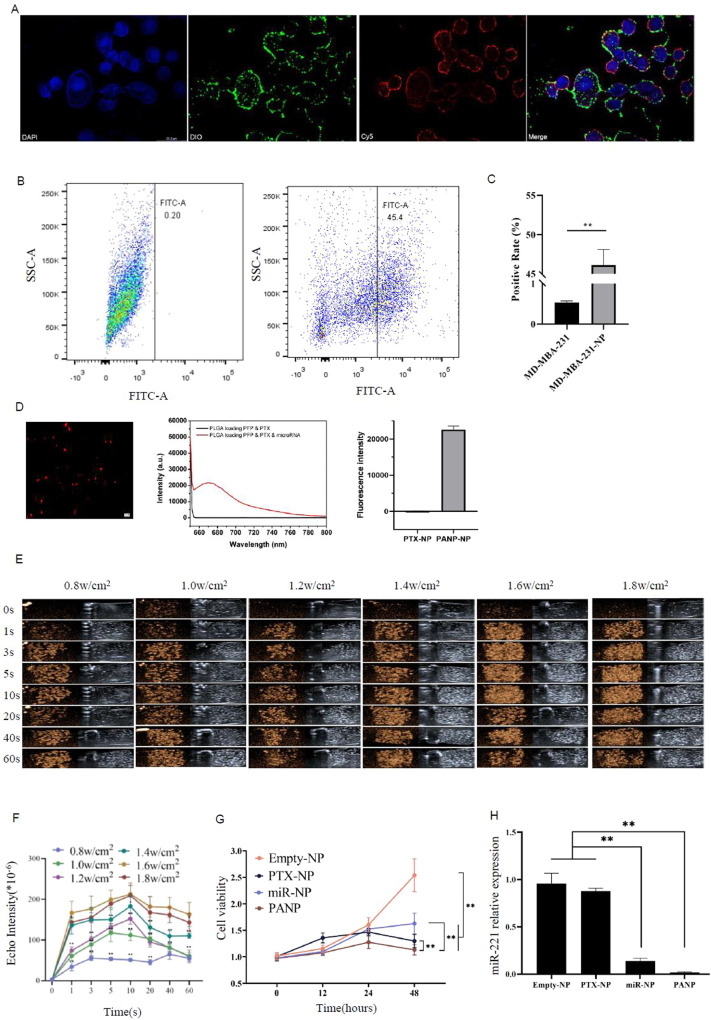
Tumor-suppressing function of PANPs in MDA-MB-231 cells. **A** PANP internalization into MDA-MB-231 cells after 6 h of co-incubation. DAPI for cell nucleus, Dio for nanoparticles, and Cy5 for small RNAs in PANPs. **B** Flow analysis of Dio fluorescence in MDA-MB-231 cells with or without incubation with PANPs. **C** Quantitative analysis of **B**. **D** After we encapsulated the miRNA after cy5 staining in NP and internalized it with MDA-MB-231 cells cells, we detected it using a fluorescence spectrophotometer. **E** Ultrasound images of PANPs in MDA-MB-231 cells before and after sonication with different intensities of 0.8-1.8 W/cm^2^ for different times from 0 to 60 s. **F** MDA-MB-231 cells carrying Empty-NP, PTX-NP, miRNA-NP, and PANP were treated with 1.6 W/cm^2^ for 60 s and cultured for 0, 12, 24, and 48 h, and cell viability was detected (*n* = 6 independent experiments). **G** Different types of nanoparticles (NP) were co-incubated with MDA-MB-231 cells, followed by an ultrasonic burst. Cell viability was assayed at 12, 24, and 48 h after sonication. **H** QRT-PCR analysis of the miR-221 level in MDA-MB-231 cells treated with different types of NPs in **F**. Data are presented as the mean ± SEM (*n* = 3). **P < 0.01.

The florescence-labeled PANPs were co-cultured with the MDA-MB-231 human breast cancer cells for 6 h, followed by DAPI staining of the cell nucleus, DIO staining of the nanoparticles, and Cy5 staining of anti-miRNAs (Fig. 3A). As a result, PANPs were internalized by the MDA-MB-231 cells and enriched in the cell membranes. A PANP intake rate as high as ~45% was confirmed using DIO-positive flow cytometry analysis (Fig. 3B, C). In order to determine the loading efficiency of the anti-miRNA inhibitor in the PANPs, a Cy5-labeled small RNA control was assembled and applied for fluorescence detection (Fig. 3D). The effects of PANPs on the cells were analyzed using different ultrasonic intensities (0.8–1.8 W/cm^2^) for different times (0–60 s) (Fig. 3E). As shown in Fig. 3F, echo signal intensities showed a time-dependent increase pattern within the first 10 s, gradually declining after that. Sonication with 1.6 W/cm^2^ for 10 s was demonstrated as the best ultrasound imaging condition in vitro.

In order to determine the ability of PANPs to suppress cancer cells, different types of nanoparticles containing PTX or anti-miR-221 or PTX+anti-miR-221 were applied to treat MDA-MB-231 cells, followed by sonication with 1.6 W/cm^2^ for 60 s to burst the PANPs in vitro. Cell viability was assayed at 12, 24, and 48 h after sonication. As shown in Fig. 3G, all three types of nanoparticles containing PTX or anti-miR-221 or PTX+anti-miR-221 significantly suppressed the cancer cell proliferation compared with empty nanoparticles as control. Notably, the PANPs containing PTX+anti-miR-221 showed higher efficiency in suppressing the viability of MDA-MB-231 cells than PANPs containing PTX or anti-miR-221 alone (Fig. 3G). As expected, the miR-221 levels in MDA-MB-231 cells showed significant suppression by nanoparticles containing anti-miR-221 (Fig. 3H).

### Tumor-homing capability of PANP-RAWs

**Fig. 4:**
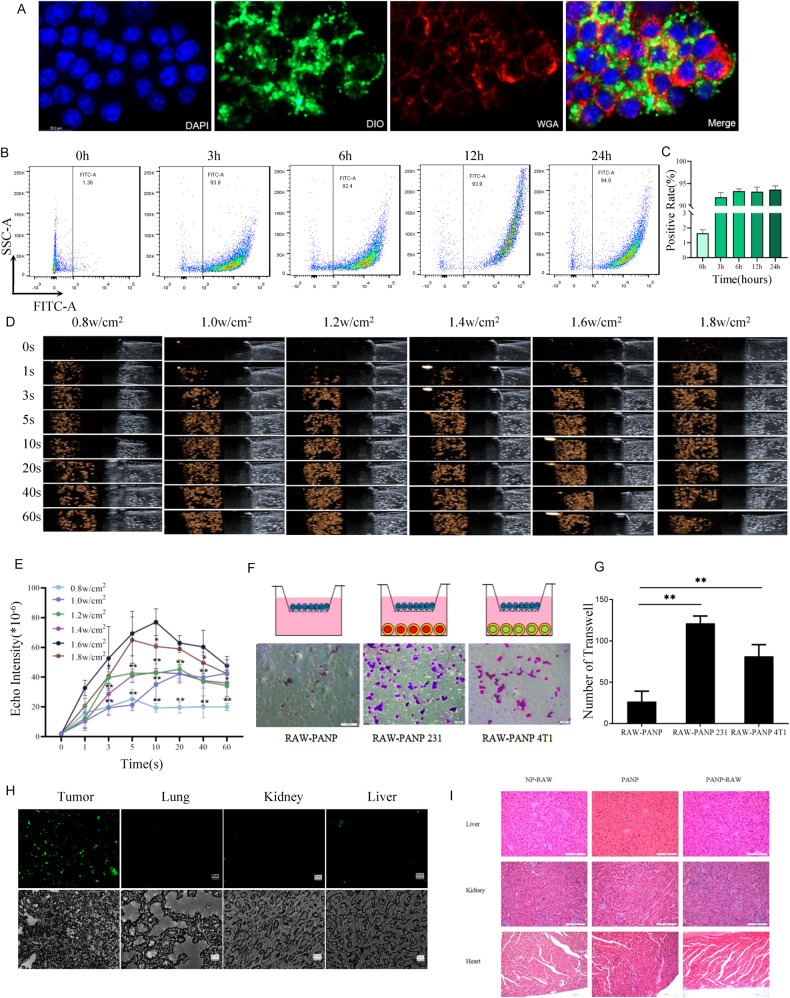
Tumor-homing capability of PANP-RAWs in vitro and in vivo. **A** PANP intake by RAW264.7 cells. DAPI for the cell nucleus, DIO for nanoparticles, and WGA for the cell membrane. **B** Flow cytometry analysis of DIO fluorescence in RAW264.7 cells after incubation with PANPs for 0, 3, 6, 12, and 24 h. **C** Quantitative analysis of **B**. **D** Ultrasound images of PANPs in RAW264.7 cells before and after sonication with different intensities of 0.8–1.8 W/cm^2^ for different times from 0 to 60 s. **E** Quantitative analysis of echo signal intensities in **D**. All comparisons were vs. 0.8 W/cm^2^. **F** Transwell analysis of PANP-RAWs in the upper chamber towards the bottom chamber with or without breast cancer cells MDA-MB-231 or 4T1. **G** Quantitative analysis of **F**. **H** Fluorescence analysis of DIO in frozen tissue sections of the breast tumor, lung, kidney, and liver from the breast tumor-burden mice injected with DIO-NP-RAWs through tail-vein. **I** H&E analysis of DIO in frozen tissue sections of the breast tumor, lung, kidney, and liver from the breast tumor-burden mice injected with DIO-NP-RAWs through tail-vein. Data are presented as the mean ± SEM (*n* = 3). **P < 0.01.

We compared the inherent tumor-homing behavior of macrophages containing different contents with that of normal macrophages and no significant abnormalities were observed (Supplementary Figure S4). Given the tumor-homing capability of macrophages, RAW 264.7 cells were applied for the tumor cell-targeted drug delivery. PANP intake by RAW264.7 cells was validated by confocal micrographs (Fig. 4A) and flow cytometry analysis at 3, 6, 12, and 24 h after co-incubation (Fig. 4B, C). Different concentrations of PANPs (from 0 to 50,000 µg/mL) were applied to RAW264.7 cells for the cytotoxicity test before ultrasound stimulation. In order to determine the ultrasound-triggered vaporization of PANP in RAW264.7 in vivo, ultrasonic intensities varying from 0.8 to 1.8 W/cm^2^ for 0–60 s were applied (Fig. 4D). The echo signal intensities showed an ultrasound power-dependent pattern and reached a peak for imaging when sonicating for 5–10 s (Fig. 4E), consistent with the results in MDA-MB-231 cells (Fig. 3E).

In order to determine the tumor-homing capability of PANP-RAWs, Transwell assays were performed in vitro with breast cancer cells MDA-MB-231 or 4T1 in the bottom chamber. As shown in Fig. 4F, G, significant increases in migrated PANP-RAWs were observed when cancer cells were present in the bottom chamber. In contrast, fewer PANP-RAWs migrated through the membrane toward the cell-free medium.

The tumor-targeting capability of PANP-RAWs was validated in vivo. A breast cancer mouse model was established using human MDA-MB-231 cancer cells. RAW264.7 cells carrying DIO fluorescence-labeled nanoparticles (DIO-NP-RAWs) were applied to the tumor-burden mice through tail-vein injection. Analysis of the frozen tissue sections from the mice indicated accumulation of the DIO fluorescence in the tumors, while few signals were detected in other tissues, including the lung, kidney, and liver (Fig. 4H). HE staining and frozen sections showed that the histology of liver, kidney, and heart of the mice treated with Empty-NP-RAW, PANP, or PANP-RAW showed no obvious abnormality, indicating that the study drugs did not cause damage to the organs under the protection of the complex, which support the safety of PANP-RAWs (Fig. 4I).

### Tumor-suppressing capability of PANP-RAWs

**Fig. 5:**
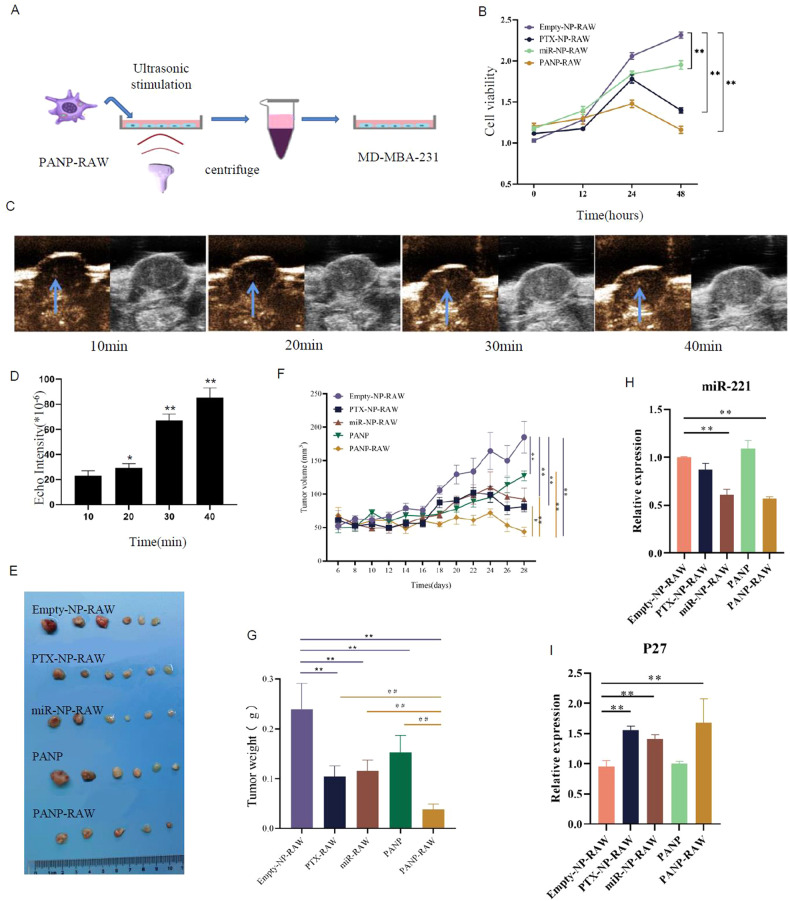
Tumor-suppressing capability of PANP-RAWs in vitro and in vivo. **A** Schematic representation of the workflow to collect supernatant of RAW264.7 cells carrying different types of nanoparticles (RAW-NPs) to treat MDA-MB-231 cells. **B** Cell viability analysis of MDA-MB-231 cells in 0, 12, 24, and 48 h after treatment with supernatants of RAW-NPs. **C** Representative ultrasound images of the central part of the tumor tissue of MDA-MB-231 tumor-bearing mice, 10, 20, 30, and 40 min after tail resting injection of PANP-RAW (blue arrows point to the area of greatest PANP enrichment. **D** Quantitative analysis of echo signal intensities in **C**. **E** MDA-MB-231 tumor-bearing mice, Empty-NP-RAW, PTX-NP-RAW, miR-NP-RAW, PANP, and PANP-RAW, were injected tail-resting every three days, and after homing tumor tissues, tumor sites were ultrasonicated, and tumor growth curves of mice were treated for 2 consecutive weeks (*n* = 5 mice per group). **F** Tumor growth curves of the breast tumor-burden mice injected with different types of RAW-NPs through tail-vein, followed by an ultrasonic burst to release drugs 24 h after each injection. Empty nanoparticles in RAW264.7 were used as a control (*n* = 6 in each group). **G** Quantitative analysis of tumor weight in **E**. **H**, **I** Quantitative analysis of miR-221 (**H**) and p27 (**I**) in the tumors of different experimental groups. Data are presented as the mean ± SEM (*n* = 3 in **B**, **D**, **H** and **I**, *n* = 6 in **E** and **G**). *P < 0.05, **P < 0.01.

We next determined the tumor-suppressing capability of PANP-RAWs upon ultrasonic burst. RAW264.7 cells carrying different types of nanoparticles containing PTX, anti-miR-221, or PTX+anti-miR-221 were sonicated with 1.6 W/cm^2^ power for 60 s to burst. The supernatants were collected and applied to MDA-MB-231 cells, followed by cell proliferation analysis (Fig. 5A). As shown in Fig. 5B, all the MD-MBA-231 cells containing either PTX or anti-miR-221 or PTX+anti-miR-221 were suppressed compared with control nanoparticles. PANP-RAWs containing PTX+anti-miR-221 showed the highest efficiency in inhibiting MD-MBA-231 cell proliferation. After PANP-RAWs were injected through the tail vein of mice, the intratumoral blood vessels could be detected by the ultrasound system at 10, 20, 30, and 40 min, and it was found that PANP-RAWs tended to accumulate at the tumor site in a time-dependent manner; the ultrasound signal could be clearly observed and it was statistically significant (Fig. 5C, D).

In order to determine the tumor-suppressing function of PANP-RAWs in vivo, RAW264.7 cells carrying different types of nanoparticles (PTX, anti-miR-221, or PTX+anti-miR-221) were tail-vein injected into tumor-burden mice, followed by an ultrasonic burst to release the drugs. Empty nanoparticles were used as a control. As seen in Fig. 5E–G, all three types of NP-RAWs suppressed tumor growth upon ultrasound stimulation. Combining PTX with anti-miR-221 (PANPs) showed a synergetic effect in inhibiting tumor growth (Fig. 5E). In addition, the macrophage administration system (PANP-RAWs) inhibited tumor growth more efficiently than PANPs alone. In Fig. 5E, one sample was missing in the PANP-RAW group because no tumors were observed anymore after treatment.

In order to validate the biological effect of the PANP-RAW complex in vivo, we constructed a mouse model of tumor metastasis. The mean weight of the mice was 17.77 ± 2.19 g. The results suggest that the NP-RAW complex had no significant effect on the vitality of the mice in each group (Supplementary Figure S5). The changes of miR-221 and p27 in the tumors of different experimental groups were examined by qPCR analysis (Fig. 5H, I).

## Discussion

TNBC has a poor prognosis [1–3], and chemoresistance is an issue [11]. This study aimed to develop a highly effective approach for the chemotherapy-based treatment of TNBC by preparing a novel kind of nanoparticles shelled with PLGA/lipid and loaded with PTX, PFP, and anti-miR-221 and delivered by macrophages. The results showed that a macrophage-carried, ultrasound-triggered, cancer cell-targeted chemotherapeutic system was developed. A miRNA-based nanoparticle can promote paclitaxel sensitivity of cancer cells, holding the potential to treat patients with TNBC, especially those suffering from drug resistance. The innovation of this study is to use macrophages to deliver nanoparticles to the tumors and then use ultrasound locally to burst the nanoparticles to release the miRNA and PTX cargo. Even though two strategies applied in the present study were already well-known [23, 25], the strategy suggested here combines these two strategies for the first time.

Macrophages have a homing capacity for the TME [26, 27]. That property was fully exploited in the strategy proposed here by loading the macrophages with nanoparticles for delivery to the TME. A previous study proposed a similar strategy based on a CpG delivery system activated by phototherapy [7]. Still, light penetration is relatively poor in deep tissues. The use of ultrasound in the present study would allow the triggering of the nanoparticles in deeper tissues. Of note, we compared the inherent tumor-homing behavior of macrophages containing different contents with that of normal macrophages and no significant abnormalities were observed.

Indeed, ultrasound has been well applied for real-time imaging due to its capability to drive the liquid-to-gas transition of the perfluorocarbon [8]. This makes it realizable to track the movement of injected cells in vivo when carrying a vaporizable liquid, such as perfluorocarbon. In addition, ultrasound sonication has shown the potential to control drug release by vaporizing liquid perfluorocarbon to numerous microbubbles within drug-carrying cells, resulting in cell disruption and cargo drug release. The present study developed a cell-based nanoparticle delivery system by encapsulating PFP and antitumor drugs into nanoparticles and intake by RAW 264.7 macrophages. After in vivo injection, ultrasound cavitation triggered the macrophage bombs to burst, leading to drug release within tumor tissues. Furthermore, ultrasound-induced cavitations can make the cell membrane more permeable, favoring the entry of drugs and miRNAs into the cells [28].

Drug resistance of cancer cells and off-target damage to normal cells are the main factors limiting chemotherapy outcomes [29, 30]. The application of nanotechnology makes tumor-targeted drug delivery possible [8]. PTX is widely used as one of the first-line chemotherapy drugs to treat patients with breast cancer [31, 32]. Unfortunately, the frequent occurrence of chemoresistance has become a major limitation of its therapeutic effect [8]. A previous study by the authors’ group showed aberrant overexpression of miR-221 in resistant breast cancer cells [18]. The targeted inhibition of miR-221 promoted sensitivity to chemotherapy in breast cancer cells [18, 33]. In order to optimize the tumor-suppressing effect of the targeted-delivery system, an anti-miR-221 was included in the nanoparticles with PTX, followed by encapsulating PFP into the core. In the present study, the PANPs could be internalized by cancer cells. The PFP contained within the nanoparticles could be induced using ultrasound to burst the nanoparticle and deliver its content to the cells. PTX alone had effects on cancer cells, but its effect was increased by the anti-miR-221 oligonucleotide. miR-221 is involved in regulating the PTX sensitivity in TNBC cells [8]. Therefore, preventing the action of miR-221 increased the sensitivity of TNBC cells to PTX.

Regarding the off-target damage to normal cells and targeting cancer cells, emerging evidence demonstrated that a cell-nanoparticle-based drug delivery system could be a promising platform for tumor-targeted therapy due to its high drug-loading capacities and the inherent tumor-homing abilities of macrophages [34]. Therefore, this study examined RAW264.7 cells as nanoparticle vehicles to directly deliver anticancer drugs to the target tumor cells. The Transwell assay showed that the macrophage migration was enhanced in the presence of cancer cells and that the targeted cells could be killed by the PANPs after the ultrasound. The in vitro results were confirmed in vivo, where the macrophage-PANPs were confirmed to target the tumors, and the efficacy of the PANPs was also observed. In contrast, few signals of the nanoparticles were observed in other organs, including the lung, liver, and kidney. Therefore, the macrophage-based drug delivery system has strong potential to make tumor-targeted delivery come true, through which the side effects of chemotherapy on normal tissue cells would be reduced or even completely avoided. Still, a limitation is that very small foci of macrophages and nanoparticles could have been missed. In addition, the small size of the tumors in the PANPs-treated mice complicated the analyses.

HE staining and frozen sections showed that the histology of liver, kidney, and heart of the mice treated with Empty-NP-RAW, PANP, or PANP-RAW showed no obvious abnormality. The cell structure and organ morphology were intact, indicating that the chemotherapeutic drugs inside the nanoparticles did not cause damage to the liver, kidney, and heart, supporting the of the therapeutic method proposed in this study. In addition, in the macrophage-free group, the presence of nanobubbles could be observed in some organs with abundant blood vessels, indicating that the nanobubble complex could clearly target tumor cells through blood vessels but not the other organs.

Of course, despite the promising results, research is still necessary before clinical trials can be undertaken. Nevertheless, macrophage-PANPs could be a way to target TNBC and overcome chemoresistance. Macrophage-mediated drug delivery offers many advantages over traditional drug delivery methods, but the heterogeneity among macrophages used to build such delivery systems has limited their clinical application [35]. Future studies should examine that issue.

In conclusion, the present study demonstrated the high efficiency of a macrophage-based, ultrasound-triggered tumor target delivery system. It provided an approach to overcoming drug resistance by the synergetic combination of small nucleic acid molecules and anticancer drugs to treat patients with TNBC.

## Materials and methods

### Animals

All animal experiments and study protocols were approved by the Science and Technology Ethics Committee of Tongji University (#TJBB002722101). Specific pathogen-free (SPF) 6-week-old female BALB/c nude mice (weighing 15–25 g) were purchased from the Silaike Animal Company (Shanghai, China). All experiments were performed in accordance with the relevant guidelines and regulations for animal use.

### Cell culture

The breast cancer cell lines MDA-MB-231 and 4T1 were originally purchased from the American Type Culture Collection (ATCC) (Manassas, VA, USA) and maintained in our lab. RAW 264.7 macrophages were purchased from the Typical Culture Collection Center of the Chinese Academy of Sciences (Shanghai, China). The cells were cultured in Dulbecco modified Eagle medium (DMEM) containing 10% fetal bovine serum (FBS) and 1% penicillin/streptomycin at 37 °C in a humid atmosphere of 5% CO_2_.

### CCK8

The treated cell lysates of each group were centrifuged at 500 rpm and the supernatant was collected. MDA-MB-231 cells were inoculated into 96-well plates at a cell suspension concentration of 1 × 10^3^ cells/100 μl medium per well, and a total of five 96-well plates were prepared. After 12 h of seeding, the collected supernatant was co-cultured with MDA-MB-231 cells for the required time and then washed with PBS solution two times. Tumor cell proliferation was detected using CCK-8, i.e., 10 μl of CCK-8 was added to each well and the last row without CCK-8 was used as a blank. The cells were incubated in a 5% C0_2_, 37 °C cell incubator for 3 h. The absorbance values were measured at 450 nm using an ELISA plate reader.

### Oligonucleotides

The oligonucleotides for anti-miR-221 and negative control were synthesized by GenScript (Nanjing, China) with 3’ end-labeling by Cy5. The sequence of anti-miR-221 was 5‘-mG*mA*mA* mA*mC*mC* mC*mA*mG* mC*mA*mG* mA*mC*mA* mA*mU*mG* mU*mA*mG* mC*mU/3’CY5/-3’. The sequence of the negative control was 5’-mG*mU*mG* mU*mA*mA* mC*mA*mC* mG*mU*mC* mU*mA*mU* mA*mC*mG* mC*mC*mC* mA/3’CY5/-3’ (m: methylated nucleotide; *phosphotiate bound) [18]. The negative control was a scrambled sequence known to target no gene.

### Preparation of PTX/PFP/anti-miR221-loaded PLGA/lipid nanoparticles

Lipid/PLGA nanoparticles were prepared through a modified double-emulsion method [23]. Briefly, 0.1 mL of an anti-miR-221 solution containing 5 nmol of anti-miR-221 oligonucleotides and 0.1 mL of PFP were mixed, and the mixture was emulsified in an ice bath by using a 200-Wn ultrasonic cell disruptor (Qsonica, Newtown, CT, USA) at 10% power (i.e., 20 W) ultrasound probe for 120 s ultrasonic at 20 W with the pulse mode of turned off for 3 s and turned on for 1 s to prevent thermal accumulation. Then the emulsion was added into the 1 mL of dichloromethane, dissolving 50 mg of PLGA, 3 mg of DSPC, and 3 mg of PTX for further 120 s emulsification using the same parameters. Subsequently, 10 mL of deionized water, including 4% PVA, was added. The mixture was homogenized using an IKA T10 basic homogenizer (Millipore Corp., Billerica, MA, USA) for 2 min at 25,000 rpm. In order to remove the organic solvent Then, 10 mL of deionized water was added to the resulting emulsion and stirred mechanically in an ice bath for 2–6 h for evaporation. After the dichloromethane was completely evaporated, the mixture was washed with deionized water and centrifuged (8000 rpm, 5 min) three times to obtain the PTX/PFP/anti-miR-221-loaded Lipid/PLGA nanoparticles. Other types of nanoparticles were prepared using a similar method. All nanoparticles were stored at 4 °C.

### High-performance liquid chromatography

In order to determine encapsulation, high-performance liquid chromatography (HPLC) (Agilent G1311B, USA) was used for the determination of nanoparticle-encapsulated PTX. First, 1 mg of the experimental sample was dissolved in 200 μL of methanol, and 70 μL of the resulting supernatant was injected into the chromatograph. The column was an Agilent Eclipse Plus C-18, 5 µm, 250 × 4.6 mm, with a detection column temperature of 25 °C, a detection wavelength of 227 nm, a flow rate of 0.80 mL/min, and an injection volume of 70 µL. The mobile phase A was methanol (chromatographic grade, Aladdin). The mobile phase B was 0.1% formic acid water. The isocratic elution was 0-10 min, 83% A. The drug content in the drug-loaded nanoparticles was calculated from the standard curve. The encapsulation rate was calculated by the following equation. Encapsulation rate (%) = amount of paclitaxel in the nanoparticles/total drug added × 100%.

The PTX release efficiency PTX was detected by (HPLC) (Agilent G1311B, USA) with the same detection method as encapsulation. The drug content in the drug-loaded nanoparticles was calculated from the standard curve, and the drug release rate was calculated by the following equation. Drug release rate (%) = amount of paclitaxel in nanoparticles/total weight of nanoparticles × 100%.

### Characterizations of nanoparticles

The structure, size, and morphology of nanoparticles were characterized through transmission electron microscopy (TEM) (Thermo Fisher Scientific, Waltham, MA, USA) and scanning electron microscopy (SEM) (Hitachi, Tokyo, Japan). The hydrodynamic diameter and zeta potential were measured using dynamic light scattering (DLS). The confocal laser scanning microscope (TCS SP5, Leica Microsystems, Wetzlar, Germany) was used to characterize the Cy5-labeled anti-miR-221 encapsulation in nanoparticles. High-performance liquid chromatography (HPLC, Agilent 1200, USA) was used to analyze the loading rate of PTX. Encapsulation efficiency (EE)% was calculated as EE% = (PTX or miRNA/PTX or miRNA initially added to NPs) × 100%.

### Immunofluorescence

RAW 264.7 cells were cultured on slides in 6-cm dishes for 12 h, followed by co-culture with nanoparticles (5 mg/mL) for 6 h. The cells were incubated with stains (DAPI for nuclei staining, DIO for nanoparticles staining, and WGA for membrane staining) at 4 °C for 1 h in the dark. The stains included DAPI (DA002, Leagene Biotechnology, Shanghai, China), DIO (D4007, US Everbright®Inc., Suzhou, China), and WGA (MP6326-1MG, MKBio, Shanghai, China). A Leica DM300 microscope was used to capture the images. The Leica Application Suite X software 3.0.0.15697 was used for quantitative analysis.

### Flow cytometry

RAW 264.7 cells were co-incubated with DIO-stained nanoparticles for different periods (0, 3, 6, 12, and 24 h), followed by flow cytometry analysis on a FACScan system (BD Biosciences, Franklin Lake, NJ, USA). After co-incubation of NPRAW cells with different contents, the macrophages were labeled with different polarization markers (M1: CD86; M2: CD206) and analyzed using flow cytometry.

### qRT-PCR

MDA-MB-231 cell cells were inoculated into 6-cm dishes at a density of 1 × 10^6^ cells/well and cultured for 12 h. The supernatants of different groups treated with UTMD (1.6 W/cm^2^, 60 s) were co-cultured with MDA-MB-231 cells for 24 h. The samples were collected after two washes with PBS. Total RNA was extracted by the Ambion kit (Thermo Fisher Scientific, Waltham, MA, USA). After reverse transcription, qPCR was performed, and miR-221 expression differences were calculated by using 5 S as an internal reference using the 2^-ΔΔCT^ method. Primer sequences (Genscript, Co., Ltd., Nanjing, China): miR-221: 5′-AGC UAC AUU GUC UGC UGG GUU UU-3′; 5 S rRNA: 5′-AGT ACT TGG ATG GGA GAC CG-3′; UR2: 5’-CTA GAT CAG CTG GGC CAA GA-3’; p27 forward: 5’- ATA AGG AAG CGA CCT GCA ACC G-3’; p27 reverse: 5’- TTC TTG GGC GTC TGC TCC ACA G-3’.

### Cell viability assay

Cells were inoculated in 96-well plates at a density of 1×10^3^ cells/well and co-cultured with different doses of PANP for 0, 1, 2, 4, 8, 12, and 24 h. A CCK-8 reagent (Dojindo Molecular Technologies, Kimamoto, Japan) was used to detect cell viability.

### Transwell assay

Transwell chambers with 8-μm pores (Corning Inc., Corning, NY, USA) were pre-coated with ECM Gel (E1270, Sigma-Aldrich, St. Louis, MO, USA) and placed in a 24-well plate. Then, 4 × 10^4^ RAW 264.7 cells were seeded in the upper chambers with serum-free medium, while the bottom wells were seeded with MDA-MB-231 cells, 4T1 cells, or DMEM medium only. After incubation for 16 h, the migrated RAW 264.7 cells were stained with crystal violet and photographed and counted with a microscope.

### Ultrasonic examination

RAW 264.7 cells were co-incubated with different concentrations of nanoparticles for 12 h and injected into a conical chamber filled with 2% agarose gel. Echo intensities were recorded using the high-frequency ultrasound Aplio 500 (Canon, Tokyo, Japan) and plotted with time. A high-frequency ultrasound imaging system (Aplio 500) was used to evaluate nanoparticle vaporization ability and contrast-enhanced ultrasound ability. Different ultrasound intensities (0.8, 1.0, 1.2, 1.4, 1.6, and 1.8 W/cm^2^) were applied [24].

### miR-221 internalization assay

Cy5-miRNA-PANP nanoparticles were prepared using Anti-miR-221 modified by Cy5. A fluorescence spectrophotometer (670 nm) was used to compare the fluorescence absorption intensity of the PANP complex containing cy5-miRNA with that of the PANP complex without Cy5-miRNA to determine the miRNA encapsulation status.

### In vivo assays

MDA-MB-231 cells (*n* = 1 × 10^6^) were transplanted into the fourth mammary fat pad of female BALB/c nude mice to generate breast tumor-burden mice. One week after cell transplantation, the mice were treated with 2 × 10^6^ RAW 264.7 cells carrying DIO-labeled nanoparticles by tail-vein injection every other day for a total of six times (*n* = 6 in each group). At 10–40 min after each injection, the nanoparticles were activated by ultrasound for real-time imaging in vivo using a UT 1041 system (Dundex, Shanghai, China) at 1.6 W/cm^2^ for 10 s. Then, the Aplio 500 ultrasound system was immediately used to record the blood flow signal in the mass after ultrasound blasting. The echo intensities in tumors were recorded. At 24 h after each injection, the nanoparticles were burst to release drugs by ultrasonic stimulation. The treatment was performed with a UT 1041 system 1.6 W/cm^2^ for 60 s. Meanwhile, the tumor volumes were measured every 2 days. Tumor-loaded and treated mice are weighed directly using a body weight scale. On day 28, all the mice were sacrificed, and the tumors were collected for further analysis. The tumors and main organs of the mice were collected and processed into frozen sections to observe the localization and dispersion of fluorescent nanoparticles.

### Histological examination

The tissue samples were routinely paraffin-embedded. Liquid paraffin wax was added to the mold, and the tissue sample to be embedded was put into the paraffin to ensure the regular position of the tissue. After replenishing a little liquid paraffin wax, it was cooled and frozen to make the paraffin wax become solid to achieve the effect of tissue fixation and then sliced using a paraffin slicer with a thickness of about 4–8 µm. The sections were placed on slides and soaked in warm water at 40 °C to fully stretch the tissues. The sections were soaked in xylene for 10 min. The xylene was replaced for a second soaking of 10 min. The sections were soaked in anhydrous ethanol for 5 min, followed by 95%, 85%, and 70% ethanol for 5 min each. The sections were washed with PBS for 5 min each time, three times. Afterward, 100 µL of hematoxylin solution was added dropwise to each section using a pipette gun and stained for 10 min. After staining, the excess hematoxylin staining solution was washed off with distilled water. Then, 1% ethanol hydrochloride was used for differentiation. The sections were rinsed with double-distilled water. A weakly alkaline blue-promoting solution was added. The eosin staining solution was added to the sections and allowed to stain for 3 min. After staining, the sections were dehydrated in a gradient using 80% (5 s), 95% (2 min), and anhydrous (2 min) ethanol. The dehydrated sections were soaked twice using xylene for 4 min each time. The sections were dried and sealed with neutral resin. The sections were observed and photographed under the microscope.

### Statistical analysis

Data were presented as mean ± standard error of the mean (SEM) unless otherwise stated. The two-tailed *t* test was used to analyze the independent samples between groups. *P* < 0.05 was considered statistically significant.

### Supplementary information

Supplemental Figures

## Data Availability

All data generated or analyzed during this study are included in this published article and its Supplementary Information files.
