# Supplementary material for: Nanoparticles carrying paclitaxel and anti-miR-221 for breast cancer therapy triggered by ultrasound
Source: Cell Death Discov. 2023 Aug 15;9:298. doi: 10.1038/s41420-023-01594-9 (PMC10427607; doi:10.1038/s41420-023-01594-9)
Supplement: Supplementary file 1 — Supplemental Figures [file 41420_2023_1594_MOESM1_ESM.pdf]

Supplemental Figure

Supplemental Figure Legends

Figure S1. Representative images of vaporization of nanoparticles in the cell culture medium before and after ultrasonic stimulation.

Figure S2. Drug safety of NPs containing different contents in MDA-MB-231.

Figure S3. NP with different contents did not cause M2 polarization compared with the control group but caused polarization toward M1. M1 macrophages are an antitumor type macrophage and will not affect the subsequent experiments.

Figure S4. Inherent tumor-homing behavior of macrophages containing different contents with that of normal macrophages.

Figure S5. Comparison of body weight of mice in each treatment group on day 28 after treatment.

Figure S1

Before ultrasound | After ultrasound

Empty-NP

PANP

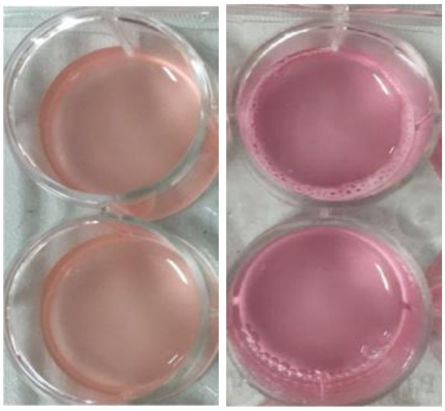

Figure S2

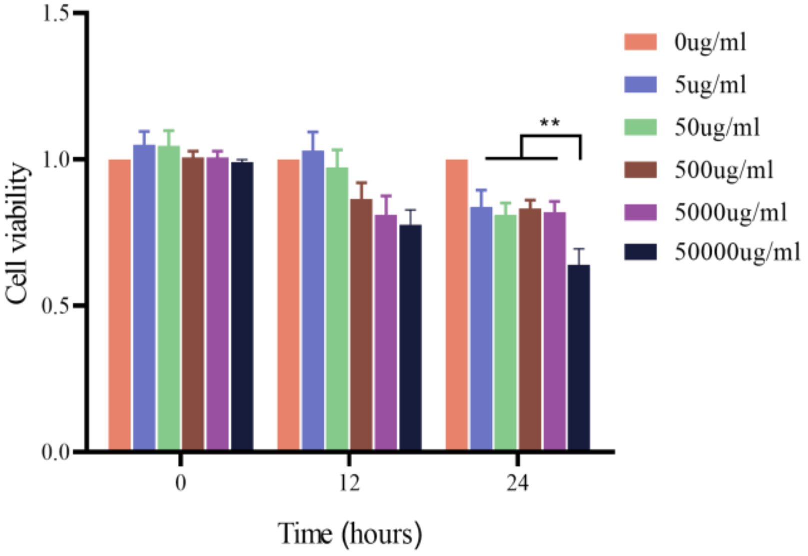
1.5
1.0
0.5
0.0
Cell viability
**
0
12
24
Time (hours)
0ug/ml
5ug/ml
50ug/ml
500ug/ml
5000ug/ml
50000ug/ml

Figure S3

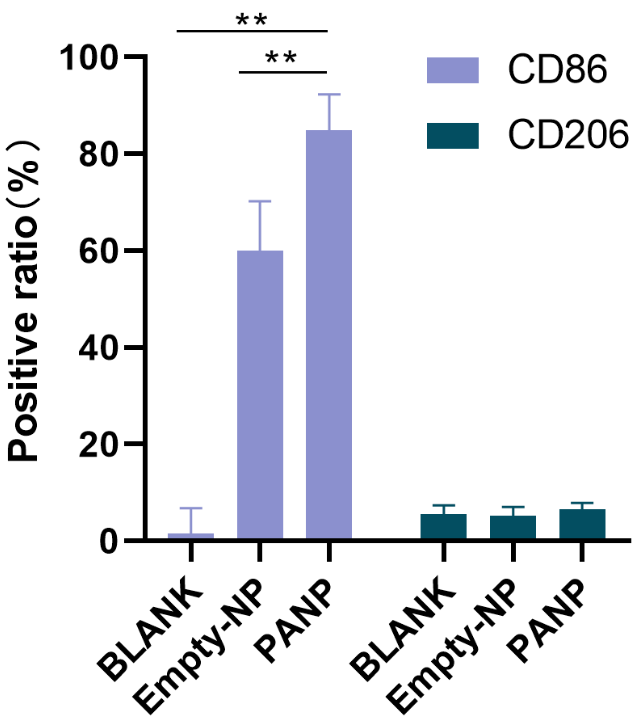
**
**
100
80
60
40
20
0
Positive ratio（%）
CD86
CD206
BLANK
Empty-NP
PANP
BLANK
Empty-NP
PANP

Figure S4

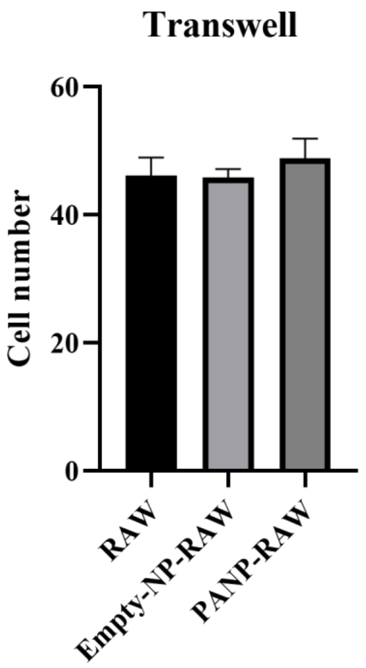

Figure S5

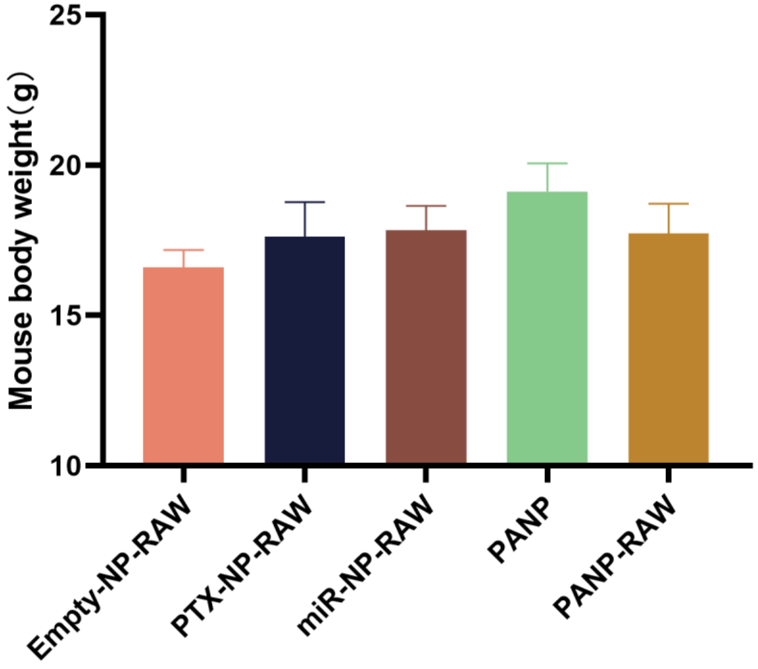

Mouse body weight（g）
25
20
15
10
Empty-NP-RAW
PTX-NP-RAW
miR-NP-RAW
PANP
PANP-RAW
